# Hermeneutic Phenomenological Understanding of the Inner Journey of Templestay

**DOI:** 10.3390/ijerph18157830

**Published:** 2021-07-23

**Authors:** Jiyoung Hwang, Hyo-Yeun Park

**Affiliations:** 1 Department of Convention Management, College of Hotel and Tourism Management, Kyung Hee University, 26 Kyungheedae-ro, Dongdaemun-gu, Seoul 02447, Korea; jy712@khu.ac.kr; 2 Department of Tourism Management, Korea Tourism College, 197-93, Ijang-ro 311, Icheon-si 17306, Korea

**Keywords:** Korean Buddhism, experiential authenticity, perceptual experiences, healing, phenomenology

## Abstract

This study delineated the templestay experience in the context of the authenticity of tourism. To understand the phenomenon of templestay experience, the study applied the hermeneutic phenomenology method. Furthermore, the researcher tried to approach the experience with an open perspective in observing essential meanings and lived phenomena. From the hermeneutic guide, the study discovered “structure of perceptions (situation, emotion, thought, action),” “perceptual experiences (rational experience, sentimental experience, judgmental experience, experimental experience),” and “experiential authenticity (superficial authenticity, situational authenticity, relational authenticity, space-time authenticity, existential authenticity)” from the lived templestay experience and developed a “descriptive model of the integrative phenomenon of templestay experience.” This study suggests the possibility of discovering new phenomena by expanding the boundaries of the perceptions of the authentic experience of tourism.

## 1. Introduction

Josef Knecht, the protagonist of Hermann Hesse’s last work “The Glass Bead Game”, wrote the following quote as he went on a journey, leaving his lifelong assignments and positions [1]:

*In all beginnings dwells a magic force. For guarding us and helping us to live.*

*So be it, heart: bid farewell without end!*

Knecht leaving his ivory tower society to accumulate various life experiences shows a journey toward “self-reflection and transcendence”, a theme Hermann Hesse constantly pursued in the coming-of-age story. Self-reflection and transcendence are the core themes in “The Glass Bead Game”, which shares the same context as the social and tourism phenomena of the 21st century modern society [2,3].

Modern society has provided a convenient life for humans, but at the same time, it has caused the problem of human alienation. Ironically, the problem leads to the human aspiration of finding one’s true self again. In the structure of society, human beings judge themselves by the eyes of others [4,5]. The image of “I” objectified by the gaze of others results in a sense of self-loss [6].

People feel skeptical about daily life being confined to the roles required by society and are pursuing tourism experience for inner transformation. By asking profound questions such as “Who am I?” and “How should I live?”, tourists want to have an opportunity to recognize and reflect on what they have lost in their daily life through spiritual enhancement tourism.

This type of tourism relates to the academic theme of authenticity as discussed in the tourism field. It is considered as consistent with authenticity because the primary purpose of such a trip is to find the value of one’s true self and feel joy through enlightenment. The concept of authenticity has had a dominant role in tourism for decades [7,8,9] but has been in dispute due to its conceptual elusiveness in tourism practice [10,11,12,13,14,15]. Accordingly, there are various perspectives on authenticity [16].

Given the authenticity related studies dealt with in the tourism field, topics such as whether tourism objects have authenticity (genuine) or whether tourists have ever felt an existential self through tourism activities are predominant [14,17]. Previous studies on authenticity tended to focus on tourists’ experience of authenticity of tourist attractions or experience of constructivist authenticity when experiencing symbolisms, such as images, expectations, and preferences projected on tourist attractions [14,17,18]. Subsequently, Wang [14] presented the concept of existential authenticity, which has been actively studied by researchers trying to explain the phenomenon of tourism in postmodernism [5,18,19,20].

Drawing from a concept of existential authenticity, the present study examines the phenomenon from an insider’s perspective (emic) and reflects on the hidden truthful reality based on the tourism experience and authenticity. Tourists, as actors, signify their experiences and reveal the essence of authenticities through their actions. To derive in-depth and meaningful phenomena of tourists, it is essential to study and observe individual behaviors as well as subjective and conscious aspects in the various contexts experienced by tourists. Therefore, this study is an ontological and epistemological inquiry to identify the essential meaning of the templestay experiences for tourists. A phenomenological methodology is presented as an approach to find this phenomenon.

Templestay aims to provide tourists with opportunities to understand and experience various Buddhist cultures and temple life while staying in a temple. It is also a form of experiential tourism that allows visitors to experience religious rituals and self-heal by overcoming their own problems through reflection on their bodies and minds. In particular, templestay is an experience composed of spiritual and cultural activities, which are essentially different from activities that pursue hedonistic values [4,21]. Therefore, it conforms to the modern tourism paradigm, which aims at offering a healthy quality of life through experiences such as relaxation and meditation.

The purpose of this study is to understand the phenomenon of templestay experience and analyze the structure of the perception of templestay authenticity and experience. Specifically, it aims to understand (a) the phenomenon of templestay experience within the framework of hermeneutic phenomenology methodology, (b) the perception structure of templestay participants, and (c) the essence of the authenticity structure of templestay experience.

## 2. Literature Review

### 2.1. Templestay

Religious tourism aims to find religious faith in the past, but modern religious tourism does not only endeavor to find religious faith. The motivations for visiting religious places consist of culture, tradition, spirituality, scenery, and so on, and all these play an essential role in making decisions to travel to religious places [22,23,24]. This fact means that templestay has also become a multifunctional tourist destination visited by tourists with various interests, including Buddhist history, heritage, culture, architecture, and experiencing the life of monks, as well as religious beliefs [25,26].

Korean Buddhism has greatly influenced and contributed to national history as a guide to national spirit or a source of national culture for more than 1700 years. In Buddhism, the inner value of finding peace of mind and eliminating suffering is more important than satisfying material desires. These Buddhist doctrines and cultures have played a role as a way of life and teaching not only for the sake of Buddhists but also for non-Buddhists [4,25].

Korean temples are not only objects of religion but also complex tourist resources that are embedded in the same place as nature and history. Most temples in Korea have beautiful natural sceneries and 80% of cultural assets, including national treasures, which are a combination of tourism and religious experiences. The geographical benefits for significant temples located in the fullness of nature also trigger tourism motivation for recreation and healing purposes [4,21,25,27].

As an official tourism product, templestay started to solve accommodation shortages for foreign tourists during the 2002 Korea–Japan World Cup. To provide tourists an opportunity to experience the life of Buddhist practitioners and learn the various aspects of Korean Buddhist culture while solving accommodation shortages, the Promotion Committee of the Year of Visiting Korea and the Jogye Order of Korean Buddhism conducted a templestay project for a total of 33 temples in 11 regions nationwide [26]. Since then, foreign interest in the unique traditional temple culture of Korea has increased through templestay operation during subsequent international events, such as the 2002 Asian Games. Accordingly, the central government and related organizations began to recognize the need to revitalize templestay as a representative cultural tourism resource [26,27].

Templestay is gradually reinforcing its status with unique programs that allow participants to experience mental and physical rest and spiritual activities beyond just lodging [28]. Currently, it is considered a traditional cultural tourism product that directly allows people to experience the traditional Buddhist culture and temple culture of Korea. It has been recognized by the OECD as a global tourism product representing Korean culture [29,30]. Templestay is attracting attention internationally because modern society needs new spiritual values to advance into a healthy society.

What is the critical value of the templestay experience to participants? Templestay-designated temples are in harmony with nature. Even without mentioning the Buddhist doctrine that emphasizes the coexistence of nature and life, the essential charm of templestay is that it is possible to center mind and body through communion with nature, away from the busy city life. Participants interact with nature during their stay at the temple [31,32,33].

Interpersonal interaction is also an essential element of experience in templestay. Social interactions occur in shared experiential activities, and positive benefits can be gained through interactions with people such as monks and other participants. During templestay, rather than experiencing self-competence or self-expression through interpersonal relationships, participants can feel psychological communion and companionship through interaction [34].

Moreover, templestay provides an opportunity for self-reflection to understand other people. In this regard, participants can gain stability, peace of mind, and internal growth. Unlike self-esteem, self-identity, and self-competence that appear while experiencing specific skills or abilities, it is understood that internal growth is achieved through self-reflection and transformation [34,35].

Templestay allows participants to have emotional experiences as well. Templestay experience is fundamentally different from activity-oriented leisure tourism pursuing hedonic values. Templestay, consisting mainly of meditation and spiritual-cultural activities, can be close to a relaxation-seeking experience. In general, relaxation-seeking experiential activities provide emotional experiences related to psychological stability, such as relaxation and comfort [4]. Templestay produces positive emotions by enabling experiences such as a sense of deviation, affinity, and understanding of the relaxation state.

In the stage of existential inquiry, the researcher reviewed templestay experience literature to enhance the understanding of the templestay experience phenomenon. This review helped gain new insight into the meaning of experience for templestay participants.

### 2.2. Tourist Experience and Authenticity

Authentic experience can be used in two distinctive ways. The first authentic experience is the authenticity experienced outside of oneself, such as culture, people, places, and objects. The second authentic experience is authenticity derived from feelings or actions that make you feel your experience is genuine, real, and sincere [16].

Modern people who experience human alienation in their daily life pursue authentic experiences, which are the main tourist motives of modern society [11]. Modern tourists are not very interested in the authenticity of tourist objects, but rather try to find their true selves through their actions or the objects they encounter while sightseeing [15]. Therefore, when dealing with authenticity in modern tourism, it is crucial to approach it through an emic perspective.

The study of authenticity in the tourism field began with Boorstin’s [10] criticism that tourism is full of fake events. Later, MacCannell [12] applied staged authenticity to tourism, and Cohen [11] categorized tourists according to how they pursued authenticity. Wang [14] presented the concept of existential authenticity by paying attention to the authentic experience of tourists. He classified three types of authenticities in tourism experience into (a) Objective/objectivist authenticity, (b) Constructive authenticity, and (c) Existential authenticity (Appendix A Table A1). He stated that tourists’ authenticity through their experiences and activities is existential. Existential authenticity is composed of personal and intersubjective emotions triggered by tourists during the tourism process [15]. Wang intended to reject the concepts of objective and constructive authenticity and establish the concept of existential authenticity. However, it is the most widely accepted theoretical framework of conceptual typology itself, regardless of whether or not the concept of existential authenticity is adopted [36].

In general, existential authenticity is experienced self-loss while living in the social role required in modern society and is used as a concept of opposition to this self-loss. It refers to the state of existence at the moment when an individual calms down toward themselves [14,37]. Sartre [38] regarded the way people live and their meaning to experience as true reality without putting existential authenticity in reality itself.

In this study, the researcher focused on whether the participants were immersed in the existential state of being during templestay rather than focused on the authenticity of tourism objects. The authenticity felt by the templestay participants in this state was defined as existential authenticity. This means the participants have an authentic experience through their subjective perception and process. Importantly, existential authenticity emerges as a diverse phenomenon because it weighs on the experience of an individual and can have a different interpretation depending on who interprets the experience [18,39].

In line with the studies related to authenticity in the tourism field, most of the studies focused on establishing the concept of authenticity. Recently, however, there has been a tendency to carry out research in connection with the authenticity experience. An analysis of previous studies shows that motivation and cultural capital [40,41], cultural resources [42], measurement of the authenticity of tourism destinations [43], and demographic characteristics such as gender and place of origin are related to authenticity. In addition, there are studies on the experience of authenticity targeting cultural heritage tourist sites [44,45].

## 3. Methods and Materials

### 3.1. Phenomenology and the Structure of Perception

This study aims to reflect on the existential authenticity of tourists who experience templestay by revealing their concealed existence in standardized human conditions through the naturalistic inquiry research method. This study begins from the ontological gaze of Heidegger’s (1962) philosophical proposition that “The ‘essence’ of Dasein lies in its existence” and examines the meaning of existence as “experience and authenticity” [46]. Consequently, a phenomenological methodology is presented as an approach to find this phenomenon.

Phenomenology appeared to be free from the positivism and reductionist viewpoints that dominated Europe in the 19th century. Husserl [47] criticized scientific positivist studies for bringing a crisis to academics and humanity by excluding the study of the meaning of human existence. He attempted to find the phenomenon directly experienced by the call “to the things themselves (Zu den Sachen selbst),” which moves away from the abstract way of thinking [48]. Therefore, phenomenology places importance on exploring the universal meaning of reality (i.e., the essence of phenomena) that is vividly given to consciousness generated by experience rather than actual events [49].

Since then, phenomenology has continued its philosophical tradition through Heidegger, Sartre, and Merleau-Ponty. Phenomenology is actively applied in sociology, psychology, nursing, medicine, and education and is concerned with constructing reality from experiences as “being-in-the-world” (in-der-welt-sein).

Phenomenology explains that human consciousness is always directed somewhere, and because of this orientation, we have a direct relationship with world experience. Here, the world is not separated by the subject or object but reveals an inseparable correlation. This is described as intersubjectivity. The underlying correlation between objectivity and subjectivity means that objectivity corresponding to a common essence can be identified in subjectivity [49]. This process, which does not take place without reflection, informs us that our existence can be observed concretely and that the meaning of existence is connected with others to provide a perspective. Therefore, understanding the world is basically from a lived experience based on the body, and it is highly interpretive and contextual in the world [50].

Human perception has been studied countless times in philosophy, psychology, and religion, regardless of East and West. Philosophical studies dealt with a matter of cognition through the rationalism of Descartes and Spinoza, the empirical theories of Locke and Hume, and the ideology of Kant, who studied the matter of cognition in depth. In psychoanalysis, a field of psychology, Jung’s theory represents epistemology [51].

In the field of religion, Buddhism explains in detail the structure of human perception. Discussions on the cognitive structure began in early Buddhism; Buddha placed importance on direct experience through sensory organs and presented the five aggregates* as the cognitive structure in which such experiences are made [52,53]. The five aggregates prove that there is no fixed, permanent “self” by showing the structure in which human beings recognize the self and eliminate human obsession [54].

* The theory of the five aggregates, or “pañca-khandhā,” is an excellent Buddhist classification system that analyzes human existence. In this system, human existence is divided into five components: form, feeling, perception, mental formations, and consciousness. Given that the five aggregates constantly change, they are impermanent and therefore bring suffering. There is no “self” in these five impermanent aggregates (non-self or anatman). A human being is only a temporary collection of these five aggregates. As such, there is no single component of the five aggregates that can be claimed as “self,” but sentient beings continually hold on to their belief in “self.” This belief comes from the five aggregates of attachment.

[55]

Heidegger [56] stated that existence is a possibility. He denied Plato’s theory of ideology, which represents Western metaphysics, and argues that the existence of human beings constantly changes and flows as they relate internally and externally with the world. Here, we can find the similarity of reason between Heidegger and Buddhism about “being” [57].

As aforementioned, explaining the structure of perception in Buddhism is not to prove that there is a fixed “self.” This explains that existence means change and possibility because human existence can be explained as the five aggregates that compose perception, but it cannot be explained with only one element. This is where Heidegger and Buddhism’s epistemology share commonality [54].

Buddhism and Heidegger share the premise of the reason for existence, but in examining the perception structure, the karmic existence of Buddhism suggests a concrete and systematic structure of perception instead of the philosophical logic of Heidegger. Therefore, it is believed that karmic existence can be used as a tool to examine the cognitive structure of templestay participants.

The present study identifies the phenomenon of templestay experience and analyzes the structure of perception of templestay authenticity and experience by asking, “What is the adverse reaction to daily life?”, “What does the templestay experience mean in modern society?”, and “How is the meaning structure of the authenticity of the templestay experience expressed?” through the research method of naturalistic inquiry.

The interpretive world is merely the world experienced by the human subject and does not coincide with the world as an object. With an individual’s interpreted world existing as the world experienced by oneself, the experience is utterly right for them [31]. Therefore, the templestay experience interpreted by templestay participants based on their own perception can be related to authenticity. Accordingly, the essence of this study is to explore what the experiences of templestay participants are and how the experiences of authenticity are expressed.

### 3.2. Data Collection and Data Analysis

Interview is the method of data collection in phenomenological methodology [58]. An in-depth interview, which can express in-depth and rich personal experiences, was selected for data collection. Interviews were conducted over six months from June to December 2016. The researcher visited the temple—the study site—twice for participant observation and conducted interviews with 13 Korean participants. The interview time ranged from 60 min to 90 min for each interviewee. Among the participants, 11 experienced templestay located in remote mountain areas and two went to templestay in urban areas.

A semi-structured interview was used to conduct in-depth interviews more efficiently as it can capture in detail the individual and comprehensive experiences of the study participants [59]. Rather than maintaining a neutral attitude in the research process, the researcher may show interest in the response content or request a complementary explanation depending on the situation. The researcher also collected data through participant observations in an attempt to increase the insight and validity of the study. The researcher participated in the Templestay Preliminary Manpower Training Course conducted by the Korean Buddhist Culture Project Group to qualify as a templestay researcher.

Participants were selected by purposive sampling according to the principles of relevance and sufficiency. Relevance means selecting participants who can provide the best information about the research, and sufficiency means collecting data to reach saturation for a sufficient and rich explanation of the research phenomenon [59]. For the phenomenological approach, participants who have experienced templestay and could reflect on their experiences were selected. Specifically, participants who showed interest after hearing the explanation of the purpose and content of this study were primarily selected. Among them, those who had a clear purpose for templestay, such as healing and religious reflection, and recognized that they experienced a different experience from their daily life during the templestay were selected. Participants who were able to make accurate statements about their experiences were also chosen. All agreed to participate in the interviews.

To increase the reliability and validity of the research and widen the boundaries of the researcher’s perception, the researcher participated in the templestay site, conducted a direct interview, and at the same time, conducted participant observation to collect data. The interview was stopped until the contents of the interview were repeated, appropriate, and sufficient [31,58,59]. The characteristics of the study participants are shown below in Table 1.

The concept of reliability in qualitative research is not a matter of study order and repeatability of results. Rather, it is a question of whether a consistent result can be found with the collected data and whether the meaning can make sense when the research results are applied to the same phenomenon [60].

This study focused on data collection and recording to increase its reliability. First, the researcher studied interview guidelines, methods, and interview question design in advance to acquire expertise and then applied and checked these issues while interviewing. Second, to improve the quality of data collection, the researcher recorded and transcribed the interviews of the participants. Their behavioral language was also written in a notepad during the interview to prevent distortion of the data. Finally, systematic and thorough research procedures were secured by applying hermeneutic phenomenology methodology, and through this, reliability was secured in research data collection and interpretation.

Establishing a direct relationship between the researcher and the research subjects (participants) is crucial in qualitative research to induce an intimate, dynamic, and lasting relationship [61,62]. To anticipate and cope with the ethical problems arising from this relationship, the researcher gained familiarity with the essential matters of research ethics in advance. In this study, the following matters were explained prior to the study being conducted. First, it was explained that the statements of all participants would not be used except for the purpose of this study. Second, for the anonymity of participants, numbers were used instead of their names. Third, participants were assured that the data collected during the research process (recording files, transcripts, memos, etc.) would be used only for research purposes. Fourth, all in-depth interviews were conducted by considering the place and time the participants would feel comfortable. Finally, as a qualitative researcher, ethical considerations included the appropriateness of the citation of research results or interview materials so that the appropriateness of the research participants’ statements and analysis of results were confirmed.

The research procedure of hermeneutic phenomenology was applied to understand the essential meaning of the phenomenon [63,64]. First, after listening to the recorded file, the researchers transcribed and revised the manuscripts several times. For unclear contents, the researchers confirmed the manuscripts from participants by telephone. Second, the accuracy of the contents of the manuscript was secured by referring to the notes taken in the templestay sites. Third, through reflective analysis, the meaningful statements were developed from the participants’ narratives. Fourth, the subject statements were changed into implicit terms by focusing on the participants’ responses to the templestay experience. Fifth, the essential themes were developed with more abstraction forms after several revisions. Finally, the researcher compared and reviewed the essential themes and previous studies and reflected them to determine the meaning and essential theme of the templestay experience.

## 4. Results

### 4.1. Recognition Structure of Templestay Experience

During the interviews with templestay participants, they perceived and shared their experiences in various ways. As more interviews were added, it was found that the participants perceive their experiences in four significant ways: (1) some described their experiences by focusing on the emotions they felt through the experiences, (2) some recognized their actions during the templestay and narrated their stories, (3) one participant judged and explained their experiences according to profit and loss, and (4) others told their narrative depending on whether or not their experience was in line with their judgment value. Heidegger [56] said that being is “to reveal oneself,” and the study participants added meaning to their existence by revealing their experiences from four perspectives.

As described above, the four types of perception were named and explained based on the Buddhist five aggregates that can best explain the cognitive structure found in this study, namely, (1) situation-oriented perception, (2) emotion-oriented perception, (3) thought-oriented perception, and (4) action-oriented perception.

These four perceptions could be found in each participant, but they showed a strong tendency to interpret their experiences specifically through one of the four cognitive elements. In other words, there was a difference in the way that the participants perceived their experiences.

#### 4.1.1. Situation-Oriented Perception

Situation-oriented perception is the “form,” “matter,” or “material form” of a being or any existence. It is in line with the same context as the world of desire and the world of objectivity. The law of profit and loss runs this world. Participants with this situational awareness tended to act according to profit and loss, and when they judged that the action was beneficial to them, they felt their experience was meaningful. Therefore, this phenomenon of the experience of perception was named “rational experience.”

Templestay participants with this perception described that their templestay experiences helped them drop away from their ego-mind and sustain a certain amount of time being happy when they return to secular society. In addition, they showed a willingness to experience the new templestay program and a positive response when asked about their intention to revisit. It also led to the act of recommending and encouraging acquaintances to participate in templestay.

*I could reset my ego-mind, and it helps for a while.*

(Participant 8)

*I live happily for a year when I go back to the Mahamudra practice.*

(Participant 10)

*Next time I go to templestay, I want to join a meditation program. Meditation is one of the Zen practices, so I want to participate in a new program.*

(Participant 5)

*I think I would recommend templestay. First, it is a different experience, and I think you could experience a transformed and awakened state. Or you can just go for healing or just take a break.*

(Participant 7)

*I want to recommend templestay to people who are suffering in their life.*

(Participant 9)

On the other hand, if the participants thought that the templestay experience was not profitable in any respect, they hesitated to revisit or expressed their intention not to revisit the templestay.

*I felt that something was missing in the templestay program. Something essential to experience my true self.*

(Participant 4)

*“Who am I?” is the question I seek to answer, but I could not find the answer during the templestay. I felt like it was taboo to ask such a question. After that, I didn’t go to templestay.*

(Participant 6)

#### 4.1.2. Emotion-Oriented Perception

Emotion-oriented perception belongs to the “sensation” or “feeling” spoken in the five aggregates and comprises a world of emotions and subjectivity. The laws of positive and negative feelings run this world. Participants in the study interpreted their experiences according to the emotions they reacted to when they encountered situations at the templestay. Therefore, the phenomenon of experiencing this perception was named “sentimental experience.”

Emotion-oriented participants described their narratives with feelings and emotions. Even when they were immersed in a specific moment, they described their emotional state by comparing the situation to an image or sound. They also stated that the moon seen from the city and the moon seen from the temple is the same, but they will revisit the temple because they feel that the moon they experienced was more beautiful. They also described themselves with feelings and explained the feelings that they experienced, as stated below:

*When I hear the Korean Buddhist Temple Bell, for example, when a pebble dropped into a pond caused a circular ripple, I could feel the sound in my head. It’s like a crystal clear sound that made my mind awakened. This image and sound are as clear as ever. I loved it.*

(Participant 1)

*I spent time alone at night, and it was definitely a view I saw during the day, but it felt different. Everything looked pretty, even the moon too. Ah! This is it. This is the reason I come to the temple. I felt overwhelmed.*

(Participant 11)

*I felt comfortable and calm. Should I say it is somewhere between calm and melancholy? That was the time to reflect on my daily life and my ego-mind.*

(Participant 2)

#### 4.1.3. Thought-Oriented Perception

Thought-oriented perception belongs to the function of consciousness, which forms concepts, perceptions, and symbols and is a world of will. Will means the importance of the value of something or action, so the laws of right and wrong run this world. Participants with a thought-oriented perception interpreted their experiences according to right and wrong. They felt their experiences were meaningful when their actions were judged to be right and virtuous. Therefore, the phenomenon of experiencing this perception was named “judgmental experience.”

During their templestay experience, the participants with this perception recognized that it was their behavior to judge someone by their standards and had time to self-reflect on what they had done. Therefore, they shed tears and truly repented when they became aware of their judgmental process. They likewise felt free and liberated, accepting that human perspectives are diverse and that those views are not right or wrong but only change depending on the perspective.

*In the olden days, knowing knowledge (about Buddhism) was so fun that I enjoyed judging someone by it. I thought I knew everything. I should’ve sought to find mindfulness.*

(Participant 8)

*If I go there, I can spend 24 h of my own freedom. It’s short, but to me, it feels like a time when I can keep away from things that pass quickly like that. At that time, I spend time looking back and reflecting on my embarrassing and shameful actions. It’s like a mirror of reflection.*

(Participant 12)

*I went to the temple because I was in agony that I hated my colleague so much. When the chief monk held a Buddhist ceremony, I heard something that broke my perception. And then I went out and cried. I felt relieved.*

(Participant 13)

*In the past, I made a lot of judgments about my actions, and I became a lot freer about it. I’ve come to realize that the same behavior can be right or wrong because of people’s different perspectives. Bad habits cannot be changed all at once, but they are changing little by little. My favorite word is flexibility.*

(Participant 10)

#### 4.1.4. Action-Oriented Perception

Action-oriented perception belongs to “mental formations (saṅkhāra)” among the five aggregates and means intentions or behavioral needs, which are active mental functions not included in the aggregates of feeling or perception. In short, it represents the world of will. The will is the desire to achieve something, and it entails the ability to act. Thus, this world is run by the law of faith. In this regard, the participants focused on their actions, told a narrative, and reserved judgment on their actions. They only interpreted their experience as a sign of the belief that their actions would help them when they went out to the real world. Furthermore, the participants with this perception meaningfully accepted their experiences when they performed the practices in templestay. Therefore, this phenomenon of the experience of perception was named “experimental experience.”

The participants with action-oriented perceptions focused on their performance and told their narrative. At this point, they did not explain their feelings or thoughts about their actions, leaving the meaning of their actions unknown.

*When I did three thousand bows, I thought I must be crazy, but I think it’s a good experience. Because I overcame it, and I’ve done it.*

(Participant 4)

*Did I practice mindfulness? I think so. Because I did the practice. But I don’t know if it worked or not. It must have just melted somewhere in my mind.*

(Participant 6)

*After participating in templestay many times, I decided to go to a Buddhist University to study more deeply.*

(Participant 8)

The process explanation in this section provides a new theoretical framework for the structure of experience. In this regard, this study was able to derive the experientiality of the templestay recognition structure. The experience of participants did not exist in the form of Plato’s idea; instead, they interpreted their experiences with their own perceptions.

### 4.2. Templestay Experience and Authenticity

Another phenomenon was discovered during the interviews with participants, who were constantly building up the meaning of the templestay experience through a hermeneutic circle. Templestay means meeting various objects and themselves in temples and building up their own possibilities of existence. Therefore, templestay can be classified as authentic tourism.

As the interviews progressed, the templestay participants were found to have experienced not only objective authenticity while facing various symbols in the temple but also existential authenticity while questioning and facing their true selves. The types of discovered authenticity were categorized on the basis of the external and intrinsic viewpoints, which are the criteria for dividing the three authenticities (objective authenticity, constructive authenticity, existential authenticity) and the authenticity types in the tourism experience presented by Wang [14]. In this regard, the current study derived “superficial authenticity,” “situational authenticity,” “relational authenticity,” “space-time authenticity,” and “existential authenticity”, as shown below in Table 2.

#### 4.2.1. Superficial Authenticity

Superficial authenticity is a classification in which participants indirectly experienced temple culture and the life of monks, but they felt the experience as staged authenticity. The participants mainly participated in rest-oriented templestay, which means that they chose the templestay for rest rather than for practice. In other words, because their purpose was to relax in the temple, they accepted the experience as a mere representation.

*Occasionally, I participated in a Buddhist memorial service, but I think it was all about taking a rest. It was fun to see monks and temple culture close too.*

(Participant 4)

#### 4.2.2. Situational Authenticity

Situational authenticity is a classification in which participants experienced the interaction between nature and temples. This authenticity is obtained by a material object (environment). Therefore, it focuses on the experience felt through interaction with various objects in the temple.

*I really liked listening to the sound of chanting. Even if I just walk around the temple, I could hear the sound quietly from afar, and I liked this.*

(Participant 2)

*I went to templestay in the winter. Twenty centimeters of snow piled up on the roof tiles, the sound of a montak (wooden percussion instrument used for chanting by Buddhist monks), a starry night, the pond covered with thin ice! I really liked the views.*

(Participant 1)

#### 4.2.3. Relational Authenticity

Relational authenticity is a classification in which participants experienced a feeling of satisfaction from the relationship between monks and other templestay participants. It was important who they went with to the templestay and that they enjoyed meeting new people in the temple.

They recognized that the templestay experience perfectly mimics the temple life and reproduces the meaning of the temple itself. However, the most crucial thing in this representation was the relationship with others, and among them, the interaction with the monks was meaningful. This authenticity was manifested in their relationships with humans.

I went to talk with the monk. *** temple *** monk. He was the light of my life and like my real father. My family was Buddhist; that is why I went to the temple often. The words of the monks were very helpful. I shed a lot of tears too. After talking to the monk and crying about my hardships, I think I regained energy.

(Participant 8)

On the other hand, if the interaction with the monk failed during the templestay experience, no authentic experience occurred.

*I thought I could possibly talk a lot with the monks. I felt that if I went and asked for wisdom, they would give me an answer. But it did not happen. They were very busy. Really busy.*

(Participant 13)

#### 4.2.4. Space-Time Authenticity

Space-time authenticity can be obtained by a spiritual uplift or spiritual response felt through the religious activity of Buddhism and an emotional response that transcends time and space felt through the belief in an object. This authenticity may seem like a concept that is at first glance similar to existential authenticity. However, in the space-time authenticity of this study, the object became the main agent because the emotional response occurred through the object. Therefore, the authenticity was directed toward the object rather than toward the existence.

*There is a famous statue of the Guanyin Bodhisattva in that temple. She is the Buddha’s daughter. She looked down toward the sea, and her gaze seemed to care for people. So, for some reason, I felt at ease as I watched the Guanyin Bodhisattva statue.*

(Participant 7)

*Guanyin Bodhisattva. I loved that word. She is the one who listens to the voice of people. That in itself is very comforting. Please listen to this voice* *of mine. I felt I could rely on Guanyin Bodhisattva and be in the comfort zone.*

(Participant 8)

#### 4.2.5. Existential Authenticity

Existential authenticity can be obtained by the participants who asked about existence and experienced self-reflection through templestay. They tried to search for their existence through encounters with nature and symbols as well as religious acts. Existential authenticity is not experienced through external objects but is obtained in the process of returning to oneself.

*The Buddhist monk played meditation music, and I meditated while listening to it. As I was following it, I was in true samādhi (a state of meditative consciousness). I was surprised. There isn’t a moment when I don’t have any thoughts, right? But at that time, my thoughts stopped, and it was as if I had fallen asleep, but I felt myself completely. I also asked the monk because I was suspicious about my state. I felt this way. Is this correct? He said it was right. From head to toe, I could feel myself.*

(Participant 9)

*The point is being myself. People really need a conversation with themselves. We live in a busy world, so we do not see ourselves—even our actions and thoughts. So we quickly see the negative aspects of others very well, but we fail to see our trash inside and keep making excuses.*

(Participant 10)

Of the five types of authenticities in this section, the experience of templestay is strongly related to existential authenticity. This is because the participants who visited templestay have questions about their existence, and their aspiration to face themselves was higher than that of other tourists. Thus, whatever the templestay program was, they were already prepared to face and accept their existence. For this reason, templestay is a suitable form of tourism for tourists seeking existential authenticity.

### 4.3. Templestay Experience and Authenticity Perception Structure

In interpreting the phenomena of the participants, it was found that they interpreted the phenomena of experience according to their individual perception. This outcome explains that experiences are accepted and interpreted by the subjective experiences and perceptions of the individual. Therefore, the phenomena of experience can be classified according to perception. Human experience does not come from the outside to the inside but is received and then interpreted from within. In other words, external stimuli collide with the ego, and the individual does not absorb the experience. However, how the ego accepts and interprets the experience with an already perhaps formed perception is determined.

In this study, the types of experiences interpreted according to a structure of perceptions were revealed. As a result, it was possible to interpret the experience according to each perception structure and classify the authenticity type into five stages according to the subject of the experience. Specifically, the participants who interpreted their templestay were found to have experienced one of the five stages or various stages of authenticities.

These results showed that, as Heidegger [41] stated, people are ultimately related to the world (“being-in-the-world”) but at the same time build their world within (“being-in-my-own-world”). The discovery of this phenomenon has significance in tourism phenomenology from the existential perspective.

**Figure 1:**
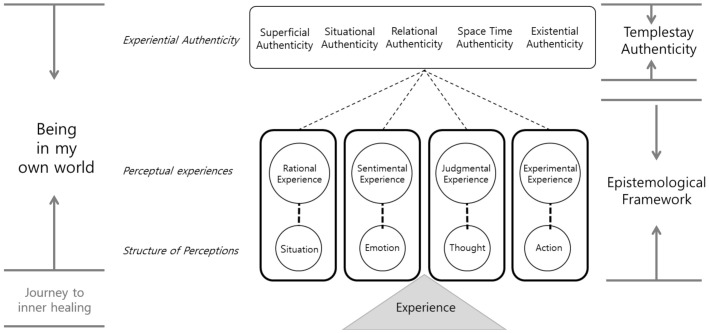
The descriptive model of the integrative phenomenon of templestay experience.

In this regard, the study suggests the descriptive model of the integrative phenomenon of templestay experience as shown below (Figure 1) in terms of the structure of perception, perceptual experiences, and experiential authenticity. The model assumes that (1) the existential experience can be analyzed from various perspectives and (2) any interpretation done in my world can be justified.

## 5. Conclusions

Encountering a true self in everyday life is not easy. Reality is a place to fill human desires. We humans are constantly moving forward to succeed, gain honor, and gain power. However, by some chance, humans question their existence and try to escape the secular life to find themselves. This study ultimately provided an opportunity to reflect on how far we are away from our underlying existence value by revealing their narratives out into the world.

The phenomenon that was notable in this study is the perception of the templestay participants who interpreted their templestay experience through their perception. Therefore, this study sought to examine the perception structure of templestay participants and present a model of how they interpret and accept the experience in their perception. The results of this study have the following academic significance.

First, this study attempted to understand the tourism authenticity experience phenomenon that has been thus far overlooked in the context of templestay. This phenomenon is analyzed by applying hermeneutic phenomenology, which was determined to be the most suitable research method to identify this phenomenon. This is because a phenomenon reveals itself as a phenomenon but is transmitted as a narrative by one’s interpretation when it is delivered. In fact, although the hermeneutic phenomenology methodology is used to research lived experience in various fields, very few research cases use this methodology to research lived experience in tourism. Therefore, this study contributed to expanding tourism research methods by applying hermeneutic phenomenology.

Second, this study is significant in that it found the perceptual structure of five aggregates of Buddhism while participants interpreted their narratives through the hermeneutic process. Therefore, this study was able to draw the perception structure of the templestay experience. The experiences of the templestay participants did not exist as shadows projected on the wall as the allegory of the cave explained. Rather, they interpreted their experiences with their perceptions.

Third, the study suggested a templestay lived experience model by examining the perceptions of participants. Templestay participants understood, interpreted, and accepted their experiences from the point of view of “I”. If this narrative about “I” is used to measure the distance from others, then it can be used to recognize the difference between “I” and others and aim for closeness rather than distinguishing oneself and others. In other words, the narrative suggests that it can be the beginning of honest communication.

Finally, the narratives of templestay participants were interpreted and identified with five authenticities, including Wang’s [14] three types of authenticities. This outcome suggested the types of authenticities according to the degree of a participant’s immersion in the templestay experience. To date, various types of authenticities have been suggested in tourism studies. These conceptual frameworks for authenticity opened up possibilities for this research. This study aimed to understand tourism (pilgrimage tourism, healing tourism, etc.) and religiously oriented tourists by discovering authenticity types based on the phenomenon of experience that emerged within the tourism field aiming for spiritual, cultural activities.

The results of this study suggest some practical implications so that templestay can faithfully fulfill the role of tourism that pursues authenticity. First, tourists who want to escape from reality searching for templestay do not simply escape daily life to enjoy themselves. The interview results revealed that templestay participants often looked for templestay to reflect on the problems they faced in reality and find their lives’ direction. Therefore, it is necessary for temples that offer templestay to think about how to fully understand and share the difficulties of templestay participants.

Second, it is possible to develop various programs based on the type of authentic experience presented in this study. Since the templestay programs currently in progress are similar for each temple, the attractiveness of templestay may decrease. Therefore, if we can develop various practice programs to experience authenticity, Koreans and people from all over the world would participate. It will be an opportunity to promote Korean Buddhism to the world and, at the same time, an opportunity for people around the world to be healed through Korean Buddhism.

Third, it is people who raise the templestay value. In particular, it was found that the competence of the supervisors and practitioners leading the templestay greatly affected the participants. Therefore, education must be provided to mentor monks and practitioners on how to deal with people from all over the world.

Templestay has developed rapidly in the past 10 years. It would not be an exaggeration to say that such development is the result of the investment and support of the government and related organizations. This study examined templestay in the context of tourism authenticity and found that the experience of templestay participants was similar to the results presented in religious tourism research [22,23,25]. At this point, where religion and tourism cannot be separated, it is necessary more than ever to discuss the phenomenon of the templestay experience so that people who participate in templestay can have a genuine experience.

Although this study derives meaningful discussions and implications through the phenomenon of the templestay experience, the following limitations and directions for follow-up research are suggested in conducting hermeneutic phenomenology research. First, this study did not consider the types of templestay programs, durations, and participants’ cultural background that may affect one’s experience. It suggests considering these factors to derive various and meaningful phenomena of the experience. Second, future empirical studies will be needed to secure the representativeness of the model found in this study and refine the theory. Third, it suggests applying folklore study to examine the lives of participants, the monks, and the bodhisattvas in the temple closely. Additionally, it would be interesting to focus on the religious phenomenon of templestay experience and discuss it from the perspective of modern religious tourism.

## Figures and Tables

**Table 1:** Characteristics of participants.

No.	Sex	Age	Job	Religion	Templestay Experience	Place of Templestay
1	F	20s	Employee	No religion	3 times	Woljeongsa, Pusoksa
2	F	20s	Between jobs	Buddhist	3 times	Woljeongsa, Pusoksa
3	F	30s	Researcher	Christian	1 time	Golgulsa
4	F	60s	Housewife	Buddhist	Over 10 times	Bongjeongam, Kilsangsa
5	M	30s	Between jobs	Buddhist	Over 10 times	Baekdamsa, Cheongryangsa, Sudeoksa, Yongmunsa
6	M	30s	Employee	No religion	5 times	Haeinseonwon
7	F	20s	Student	Christian	1 time	Naksansa
8	F	40s	Housewife	Buddhist	5 times	Silsangsa, Hwaeomsa,
9	M	40s	Self-employed	Buddhist	Over 10 times	Sudeoksa, Kabsansa, Bulgapsa
10	F	50s	Yoga instructor	Buddhist	Over 10 times	Daekwangsa, Sudeoksa, Tushita Meditation Centre
11	F	30s	Employee	Buddhist	3 times	Sudeoksa, Bulgapsa
12	F	20s	Journalist	Christian	1 time	Myogaksa
13	M	30s	Researcher	No religion	4 times	Haeinsa, Kabsansa, Myojuksa

**Table 2:** Types of templestay authenticity.

Main Agent		Type	Details
Outside object	Material object	Superficial Authenticity	Participants experienced the culture of temples and the life of monks, but they felt it as staged authenticity.
		Situational Authenticity	Participants placed great importance on their interaction with nature and the temple environment.
	People	Relational Authenticity	The most important thing for participants in their templestay experience was their relationship with others, especially their interaction with the monks.
	God (Religion)	Space-time Authenticity	It was an emotional response that transcended time and space felt through religious beliefs due to the height of the spirit or the spiritual sensitivity through religious activities called Buddhism.
Inner self		Existential Authenticity	Participants who questioned existence through templestay and experienced self-reflection were the types of people who explored their existence.

## Appendix A

**Table A1:** Three Types of Authenticities in Tourist Experiences by Wang (1999).

Type		Details
Object-related Authenticity	Objective authenticity	Objective authenticity refers to the authenticity of originals. Correspondingly, authentic experiences in tourism are equated to an epistemological experience (i.e., cognition) of the authenticity of originals.
	Constructive authenticity	Constructive authenticity refers to the authenticity projected onto toured objects by tourists or tourism producers in terms of their imagery, expectations, preferences, beliefs, powers, etc. There are various versions of authenticities regarding the same objects. Correspondingly, authentic experiences in tourism and the authenticity of toured objects are constitutive of one another. In this sense, the authenticity of toured objects is in fact symbolic authenticity.
Activity-related Authenticity in Tourism	Existential authenticity	Existential authenticity refers to a potential existential state of Being that is to be activated by tourist activities. Correspondingly, authentic experiences in tourism are to achieve this activated existential state of Being within the liminal process of tourism. Existential authenticity can have nothing to do with the authenticity of toured objects.
